# Factors Affecting Voluntary HIV Testing Among General Adult Population: A Cross-Sectional Study in Sarawak, Malaysia 

**Published:** 2020-03

**Authors:** Aren Sinedeh Lemin, Md Mizanur Rahman, Cliffton Akoi Pangarah, Andrew Kiyu

**Affiliations:** Department of Community Medicine and Public Health, Faculty of Medicine and Health Sciences, University Malaysia Sarawak

**Keywords:** Voluntary Human Immunodeficiency Virus (HIV) Testing, Sarawak, Malaysia

## Abstract

**Objective:** Voluntary HIV testing is one of the key strategies in the HIV/AIDS prevention and control program. New National Strategic Plan for 2016-2030 in Malaysia, adapt three zeros viz. ‘zero new infections of HIV/AIDS, zero discrimination and zero AIDS-related deaths’. This study aimed to determine the predictors of voluntary HIV testing in Sarawak.

**Materials and methods:** A cross-sectional study was conducted with a total of 900 respondents (450 males and 450 females) from the state of Sarawak, aged 18 years and above, who were selected by gender-stratified multistage cluster sampling. Data were obtained by face-to-face interview using a pretested questionnaire. Binary logistic regression analysis was done to determine the potential predictors for voluntary HIV testing.

**Results:** The prevalence of HIV testing was higher among female respondents (26%) compared to male respondents (14.2%), and the difference was statistically significant (p < 0.001). Binary logistic regression analysis revealed that household income more than MYR 1501 (p = 0.009), not living with a partner (p < 0.001) and discussion on HIV/AIDS (p = 0.019) appeared to be predictors for the male respondents, while, ethnicity was statistically significant for female respondents (p < 0.001).

**Conclusion:** The utilisation of HIV testing was low in both males and females. Thus, the finding of this study could be considered when designing HIV education and screening program in Sarawak.

## Introduction

Globally, there were approximately 36.7 million people with HIV/AIDS (PWHA) at the end of 2015. Moreover, an estimated 2.1 million individuals worldwide became newly infected with HIV in 2015 and this included 150,000 children mostly living in Sub-Saharan Africa. The HIV positivity appeared during pregnancy, childbirth or breastfeeding. However, 40% over 14 million people throughout the world still need to access HIV testing services (1). In Malaysia from the year 1986 to 2016, there are about 111916 total reported HIV cases with 89% of total reported cases being males, and 11% were females (2). The HIV notification rate in Malaysia showed a declining trend from 28.4 per 100000 people in 2002 to 11 per 100000 in 2016. However, the HIV notification rate plateaued from 2010 to 2016. According to the STD/AIDS Control Section in the Sarawak Health Department, the number of HIV and AIDS cases reported and notified under the Prevention and Control of Infectious Diseases Act 1988 is increasing (3). However, the exact prevalence of HIV infection is unknown, and this may be due to underreporting, underdiagnoses, and the asymptomatic manifestation of the disease.

HIV testing is an integral part of HIV prevention strategies. The proportion of testing varies across countries such as Rwanda (75%); Tanzania (55%), USA (45%) and Malaysia (20.6%) respectively (4-6). Moreover, in Malaysia, HIV testing started in 1985, and it is free in all government health facilities (7). In 2014, the Malaysian population was 30.6 million, but only 1.4 million of them tested for HIV testing resulting in the identification of 3517 new HIV cases. Half of the new cases were considered to be a late diagnosis (7). Hall et al. (8) reported that it is challenging to combat HIV/AIDS infection because several studies using mathematical model showed that 50% of new diagnosed HIV/AIDS are derived from People With HIV and AIDS (PWHA) who were not aware of their HIV status. HIV testing may have a potential effect on HIV transmission and serve as an entry point to HIV-related services such as antiretroviral treatment (9). Thus early diagnosis and treatment can be given to those PWHA and this will give good outcomes (5) namely, reduction of mother-to-child transmission (10), reduction of morbidity and mortality among PWHA (11) and improved quality of life among PWHA (10). Moreover, Cohen et al. (12) documented that PWHA receiving HIV treatment would be able to reduce new infection among serodiscordant couples by 96%.

Voluntary HIV testing was found to be associated with socio-demographic characteristics that include gender, age, marital status, education level, socio-economic status (13, 14), knowledge related to HV/AIDS (15), HIV/AIDS risk behaviour (16) and HIV/AIDS-related stigma (17). In Malaysia, stigma towards PWHA is one of the most significant challenges to control HIV/AIDS infection (18). 

HIV testing is one the of the key strategies in preventive measures to control HIV/AIDS in Sarawak, Malaysia to achieve the Three Zeros: ‘zero new infections of HIV/AIDS, zero discrimination and zero AIDS-related deaths’ in-line with the New National Strategic Plan for 2016-2030. However, there are limited studies regarding the status of voluntary HIV testing. This study aimed to determine the gender-stratified prevalence of voluntary HIV testing and the factors affecting it. 

## Materials and methods

***Study design and sampling*** **: **This community-based cross-sectional study was conducted in the state of Sarawak, Malaysia. A gender-stratified multistage cluster sampling was used to select the participants. Sarawak State is divided into 13 administrative divisions distributed geographically over three regions: northern, central and southern region. An administrative division was selected randomly from each region, and from each division, two districts were selected randomly. From each selected district, five villages were selected randomly. Then, from each selected village, 30 households were selected by a stratified systemic random sampling where an adult male and female aged 18 years and above were selected at every k^th^ number of families in the village household list provided by the ‘Ketua Kampung’ (Village headman). That is at the final stage, either one male or female respondent was randomly selected from the household which considered as male household and female household. So, 450 male and another 450 female were included in the analysis. 

***Data collection instrument***: A questionnaire-guided interview was used for data collection. The questionnaire covered HIV-related Knowledge (19); HIV test and disclosure (20), interpersonal communication about HIV/AIDS (21); media communication (22); risk behaviour of HIV/AIDS (23,24); HIV/AIDS-related stigma (1, 19) and were adapted from the sources cited. The HIV testing questionnaires were determined by asking the history of HIV testing such as, ‘Have you ever tested for HIV in your lifetime?’ 

***Data collection procedure*** ***: ***Before data collection, permission was taken from the ‘Ketua kampung’ (village headman) or ‘Tuai Rumah’ (Longhouse headman). Research assistants were trained for one-week to familiarise them with the questionnaires. Then a pilot test was conducted among 30 respondents to test whether the wordings used were clear and whether there was a need to refine the questionnaire. The Cronbach’s alpha was 0.70 and above. Data were collected by face-to-face interview by male research assistants for male and female research assistants for the female respondents. Furthermore, each respondent gave informed written consent before data collection and strict confidentiality of information and anonymity of data have been maintained. The Medical Ethics Committee of Universiti Malaysia Sarawak approved the study (UNIMAS/NC-21.02/03-02 Jld.2 (08) dated on 11^th^ February 2016; The clearance was also obtained from Clinical Research Centre, and the National Medical Research Register, Ministry of Health, Malaysia [NMRR-16-192-29374 (IIR)] dated on 31^st^ March 2016. 

***Data entry and statistical analysis*** ***: ***Microsoft Excel was used for data entry and IBM Statistical Package for Social Science (SPSS) version 22.0 (25) was used for analysis. For descriptive analysis, frequencies, means, and standard deviations were presented. Multivariate analysis was used to determine the predictors for HIV testing. A p-value of less than 0.05 was considered statistically significant.

## Results

***Socio-demographic characteristics*** **:** Table 1 illustrates the socio-demographic characteristics of the respondents. Most male and female respondents were Malay, Muslim, living with a partner with the secondary school as the highest education level. It was found that most of the female respondents were unemployed, while male respondents were self-employed. The mean age of the male was 41.57 years and the female was 38.99 years. There were statistically significant differences found in terms of the level of education, occupation and monthly household income between male and female respondents (p < 0.05). No statistically significant difference was found in ethnicity, religion, living status and family size (p > 0.05).

***History of HIV testing: ***One-fifth (20.1%) of the respondents ever tested for HIV with a median frequency of one. Some of the respondents had been tested up to seven times. Among them, 72.4% did not mention any reasons for testing, 15.5% had a history of medical check-up, and another 11.6% were tested during their pregnancy check-up. Gender-stratified analysis on HIV testing revealed that the proportion of HIV testing was found to be high among females (26%) compared to males (14.2%) counterparts and the difference was statistically significant (p < 0.001). However, the effect was small in phi-coefficient (phi-coefficient = 0.147, df = 1) (Table 2).

**Table 1:** Socio-demographic characteristics

**Characteristics**	**Male (n = 450)**		**Female (n = 450)**		**P-value**
	**Frequency**	**%**	**Frequency**	**%**	
Age (years) (mean ± SD)	41.57 ± 13.45		38.99 ± 13.09		0.004^a^
Ethnicity					
Iban	77	49.0	80	51.0	0.988^b^
Malay	200	50.6	195	49.4	
Bidayuh	73	49.7	74	50.3	
Others^d^	100	49.8	101	50.2	
Religion					
Christianity	106	49.3	109	50.7	0.705^b^
Islam	313	49.8	316	50.2	
Others^e^	31	55.4	25	44.6	
Living Status					
Living with partner	329	73.1	325	72.2	0.765^b^
Living without partner	121	26.9	125	27.8	
Median Family Size	5.0		5.0		0.716^c^
Level of education					
No formal education	76	36.2	134	63.8	< 0.001^b^
Primary	105	55.6	84	44.4	
Secondary	233	53.9	199	46.1	
Tertiary and above^f^	36	52.2	33	47.8	
Occupation					
Unemployed	63	17.2	303	82.8	< 0.001^b^
Self employed	187	75.1	62	24.9	
Government job	61	66.3	31	33.7	
Private job	139	72.0	54	28.0	
Median household income (MYR)	900.0		800.00		< 0.001^c^

a p-value reached from independent sample t-test;

b p-value reached from chi-square test,

c p-value reached from Mann-Whitney U test,

d Others included Melanau, Chinese, Org Ulu,

e Others included Buddhism, Hinduism, no religion,

f Tertiary and above included college and university level education

**Table 2:** Gender-stratified percentage distribution of history of HIV testing (n = 900)

**History of HIV testing**	**Male (n = 450)**	**Female (n = 450)**	**x** ^2^ ** (df)**	**P-value** *	**Phi/Cramer’s V**
No	85.6	74.0	19.426(1)	0.001	0.147
Yes	14.4	26.0			

* p-value reached from Chi-square test

***Gender-stratified factors affecting the HIV testing: Stepwise binary logistic regression analysis: ***All the factors which were found to be significantly associated with HIV testing, i.e. age in years, ethnicity, and living status, occupation, and household income, discussion of HIV/AIDS and content of discussion based on the Pearson’s Chi-square test of independence were further analysed using binary logistic regression. The dependent variable was dichotomized into ‘yes’ and ‘no’. A forward and backward selection method of binary logistic regression analysis was done to identify potential factors that predict HIV testing both male and female separately. The detailed model fitting information in both male and female data were presented in table 3. 

The analysis revealed that three variables were found as important predictors in the final full model (step 3) namely household income (> MYR 1500), not living with a partner, moderate discussion of HIV/AIDS matters. From the analysis, it showed that those who had a household income more than MYR 1500 per month was 2.86 (95% CI: 1.14, 7.21; p = 0.009) times more likely to do HIV testing compared to those who had a household income less than MYR 500 per month. Moreover, male respondents who did not live with their partners were 4.47 (95% CI: 2.49, 8.03; p = < 0.001) times more likely to utilise HIV testing compared to those living with their partners. Meanwhile, those who had a moderate frequency of discussion on HIV/AIDS among their community were 3.45 (95% CI: 1.41, 7.21; p = 0.019) times more likely to use HIV testing compared to those who had a good frequency of discussion on HIV/AIDS. For female data, ethnicity was found to be a significant predictor in the final full model (step 4) (Iban and Bidayuh). 

**Table 3:** Gender-stratified factors affecting the HIV testing: Stepwise binary logistic regression analysis

**Variables**	**Male**				**Female**			
	**β**	**AOR**	**P value**	**95% CI**	**β**	**AOR**	**P value**	**95% CI**
Ethnicity	NI							
Malay					-0.328	0.721	0.619	0.373,1.391
Iban					-1.315	0.268	<0.001	0.150,0.482
Bidayuh					-1.307	0.271	<0.001	0.127,0.579
Others (RC)						1		
Household income (MYR)					NI			
< 500 (RC)		1						
500-1000	-0.276	0.759	0.804	0.321,1.790				
1001-1500	0.138	1.149	0.535	0.383,3.445				
> 1501	1.054	2.869	0.009	1.141,7.214				
Living status					NI			
Living with a partner (RC)		1						
Not living with partner	1.498	4.471	<0.001	2.488,8.034				
Discussion on HIV/AIDS					NI			
Poor (≤ 1.51)	-0.451	0.637	0.060	0.338,1.202				
Moderate (1.52-4.22)	1.240	3.457	0.019	1.409,7.214				
Good (≥ 4.23) (RC)		1						
Constant	-2.366	0.094			-1.174	0.309		
Model Chi-Square (df)			48.971(6)				70.654(12)	
n			450				450	
Hosmer and Lemeshow Goodness of fit			7.099(7); 0.419				8.895(8);0.351	
Nagelkerke R Square			0.185				0.213	
Cox and Snell R square			0.103				0.145	

RC: Reference category; NI: Not included

Dependent variable = HIV Testing (Yes vs. No)

Analysis indicated that Iban ethnicity (AOR = 0.26, 95% CI: 0.15, 0.48; p < 0.001) and Bidayuh ethnicity (AOR = 0.27, 95% CI: 0.12, 0.58; p < 0.001) appeared to be significant predictors of HIV testing. Iban and Bidayuh female respondents were 0.27 times less likely to do HIV testing compared to other ethnic groups.

## Discussion

Our data showed that HIV testing utilisation among male and female respondents in Sarawak was 14.4% and 26% respectively. However, HIV testing among male respondents (14.4%) in the state of Sarawak was lower compared to a national study conducted by Wong (6) among male respondents in Malaysia (20.1%). Meanwhile, HIV testing utilisation (26%) among female respondents for this study was higher compared to Wong (6) among female respondents in Malaysia (20.9%). Overall, it was higher among female compared to male. This finding is consistent with previous studies (26-28). This might be due to the integration of HIV testing into the Maternal Child Health (MCH) services (29, 30). Another reason might be that most of the female respondents had a history of HIV testing during the antenatal check-up and this is supported by Babalola (31) and Sambisa et al. (32) who reported high rates of HIV screening. Female respondents who follow-up clinics for their pregnancies under MCH services in Sarawak, Malaysia have HIV testing. The low utilisation of HIV testing among males might be explained by their ‘masculine behaviour’ of health-seeking behaviour (33). Le Coeur et al. (34) argued that males have access to HIV testing if they are symptomatic, meanwhile, women are more likely to access HIV testing if their partners tested HIV positive or during an antenatal check-up.

Ethnicity was one of the predictors for HIV testing in Sarawak for female respondents. Iban (AOR = 0.27) and Bidayuh (AOR = 0.27) were less likely to use HIV testing compared to another ethnicity. Furthermore, this finding suggests that health-seeking behaviour also varies according to ethnicity or race of the respondents (35). Therefore, further research needs to be conducted to understand this relationship.

Average household income was one of the predictors of HIV testing among male. Male with average household income more than MYR 1500 per month were 2.87 times likely to use HIV testing compared to those who earn less than MYR 500 per month. This finding is in-line with other studies (13, 14, 16, 17). Moreover, a possible explanation for the current finding could be suggestive of a sense of ability to adopt a health-protective behaviour (HIV testing) among those with higher family income. Another explanation could be the HIV testing program may not reach poor communities (36). The current finding of average household income had no significant relationship with HIV testing among female respondents.

Living status was a significant predictor for HIV testing. Male respondents who did not live with their partners were 4.47 times more likely to utilise HIV testing compared to those who live with their partners (married or cohabitant). This is similar with the findings of Berkley-Patton et al., (4) who argued that who were single, divorced, separated, widowed were more likely to report ever having an HIV test than participants who were married or in a committed relationship. However, this finding not consistent with other studies (37-39). The present result may suggest that being male and living with their partners might not know their HIV status. Another possible explanation might be that they had the low-risk perception of HIV infection (40) and afraid to know about the positive result (41, 42).

The level of frequency of discussion on HIV/AIDS was one of the predictors for HIV testing among males. It was reported that moderate discussion on HIV/AIDS in their community might increase utilisation of HIV testing among male respondents. This was supported by Storey et al. (43). This might be due to those whoever had HIV testing spoke openly regarding HIV/AIDS, thus leading to increased acceptance for HIV testing (44-46).

Although this study tried to avoid bias in the selection of the respondents, still we encountered some limitations. Firstly, due to cross-sectional study design, resulting inability to determine a cause-and-effect relationship. Secondly, information bias might occur due to recall, sensitiveness to questions and social desirability factors during the face-to-face interview. This study did not distinguish between high-risk behaviour vs low-risk behaviour people. 

## Conclusion

The government of Malaysia has to put on the effort to scale up HIV testing, particularly in Sarawak. However, discussion on HIV/AIDS among the community can improve the HIV testing in Sarawak and need tally with ethnicity and cultural acceptability among community members. Moreover, the promotion of HIV testing through communication and discussion on HIV/AIDS need to be enhanced among those low household incomes, and those living with their partner. 
